# The Mystery of Mental Integrity: Clarifying Its Relevance to Neurotechnologies

**DOI:** 10.1007/s12152-023-09525-2

**Published:** 2023-08-21

**Authors:** Hazem Zohny, David M. Lyreskog, Ilina Singh, Julian Savulescu

**Affiliations:** 1 Oxford Uehiro Centre for Practical Ethics, University of Oxford, Oxford, UK; 2 Department of Psychiatry, University of Oxford, Oxford, UK; 3 Wellcome Centre for Ethics and Humanities, University of Oxford, Oxford, UK; 4 Centre for Biomedical Ethics, Yong Loo Lin School of Medicine, National University of Singapore, Singapore, Singapore; 5 Murdoch Children’s Research Institute, Melbourne, Australia; 6 University of Melbourne, Melbourne, Australia

**Keywords:** Mental integrity, Neurotechnology, Autonomy, Neuroethics

## Abstract

The concept of mental integrity is currently a significant topic in discussions concerning the regulation of neurotechnologies. Technologies such as deep brain stimulation and brain-computer interfaces are believed to pose a unique threat to mental integrity, and some authors have advocated for a legal right to protect it. Despite this, there remains uncertainty about what mental integrity entails and why it is important. Various interpretations of the concept have been proposed, but the literature on the subject is inconclusive. Here we consider a number of possible interpretations and argue that the most plausible one concerns neurotechnologies that bypass one’s reasoning capacities, and do so specifically in ways that reliably lead to alienation from one’s mental states. This narrows the scope of what constitutes a threat to mental integrity and offers a more precise role for the concept to play in the ethical evaluation of neurotechnologies.

## Introduction

The concept of mental integrity is playing a prominent role in discussions on the regulation of neurotechnologies. Technologies such as deep brain stimulation (DBS) and brain-computer interfaces are seen to pose a potentially unique threat to mental integrity, and a number of authors have recently debated a legal right to protect it [1–5]. This right is often formulated as a negative right to protect against unauthorised and potentially harmful brain interventions [6]. However, what mental integrity is precisely, and why it is important, is unclear. Here we consider a number of ways of understanding the relevance of mental integrity to neurotechnologies, and argue that the literature on it remains equivocal about what it is, and therefore what threats to it amount to. We suggest that the most plausible reading relates to neurotechnologies that work by bypassing our reasoning capacities, and do so specifically in ways that leave us alienated from our mental lives. Contra some of the commentators on this topic, we argue this interpretation narrows what counts as a threat to mental integrity in relation to neurotechnologies.

Etymologically, the ‘integrity’ in mental integrity relates to an individual’s mental life being in some sense whole, unimpaired or undivided [6]. The concept is typically bundled with notions like being oneself or being how one wants to be, or having a unique personality or a coherent life narrative. In that sense it clearly overlaps with concepts like identity, autonomy, privacy and rationality. This imprecision in the concept is reflected in attempts to articulate a rationale for a right that protects it. For instance, Hildt describes it as a right to control our own brain states [6], and Lavazza as protecting the “individual’s mastery of his mental states and his brain data”, with threats to it running the risk of “depriving individuals of full control over their thoughts (mental states)” [7]. More recently Lavazza and Giorgi described mental integrity as the ability to *instantiate* and *realise* one’s mental or brain states without interference, with right to it protecting us from such interference [8]. It is in that regard closely linked to the concept of cognitive liberty, which has been described as the “right and freedom to control one’s own consciousness and electrochemical thought processes” and for one “to have autonomy over his or her brain chemistry” [9]. Boire similarly talks of the right to control one’s own consciousness, and to direct one’s own mental processes [10].

As we will go on to show, what any of these descriptions amount to is ambiguous. Firstly, a jumble of terms related to the brain and mind are used, including brain and mental states and processes^1^, brain data and chemistry, thoughts, and consciousness. These all refer to somewhat different things. More importantly, what is meant by “full control” or “mastery” over one’s mental states, or control over one’s consciousness or brain chemistry, is all the more uncertain.

In what follows, our goal is to consider the most plausible interpretation of what some of the commentators in this literature are concerned about. This is by no means a systematic review attempting to capture every reference to mental integrity in that literature. While this limits the generalisability of some of our criticisms, we aim to nevertheless show a lack of a clear theory of mental integrity that can explicate its constituent parts, and concomitantly, their moral weight. Without such clarity, it is difficult to evaluate the ethics of specific neurotechnologies that ostensibly threaten it.

Before proceeding with our argument, note that this literature on a right to mental integrity is, for the most part, concerned with providing a legal rationale for it. That is, commentators are concerned with its use in regulations and in settling instances where the law is broken. We are not legal scholars, and it may be that, even if there is nothing morally significant raised by neurotechnologies beyond unsettled debates related to rationality and autonomy, a legal right that highlights certain kinds of threats to mental integrity may still be warranted. However, while we concede this possibility, it is worth noting that moral arguments are often deployed, or plausibly should be, when considering whether to recognize a certain legal right. Our focus shall be on (at least some of) these moral arguments.

## Neurotechnologies and Mental Integrity

Why have neurotechnologies sparked this debate about mental integrity? Neurotechnologies involve some degree of direct interaction between technical components such as electrodes, computers or intelligent prostheses, and the nervous system [11]. The directness of that interaction plays a significant role in the debate here, though as we will argue, what counts as “direct” is difficult to determine. But regardless of the nature of their directness, these technologies include electric, magnetic and optogenetic stimulation and recording techniques that can be administered in the brain itself or via the skull. These include DBS as well as brain-computer and brain-to-brain interfaces, which may include a multitude of brain imaging techniques. Why psychotropic drugs are not typically included in this debate is unclear, although one reason may be that, while they can surely interfere with brain or mental states, they do not record and transfer associated data.

On the treatment front, neurotechnologies have been variously deployed against Parkinson’s Disease [12], depression [13], anorexia [14], ADHD [15], and in restoring mobility among paralysed patients [16]. Beyond treatment, they have been trialled in enhancing creativity [17] and boosting cognition [18], and have opened the door to telepathy-like communication [19] and even brain-to-brain collaboration between healthy users [20].

Depending on the technique, these technologies can record signals from the brain and translate them into technical commands, and/or manipulate neural signalling. It is these two features of recording and manipulating the brain that have motivated the literature on threats to mental integrity. Numerous questions emerge: who has access to what is recorded and manipulated? Is this the beginning of the end of “mental privacy” [21]? Can we be “hacked” [22]? What will happen to our personal identity and sense of agency [23]? And many more questions [6]. At the heart of these questions is a real possibility that these technologies will have implications for our ability to control our mental lives.

Hence some of the statements in the introduction about the importance of individuals maintaining control over their brain or mental states. We noted the use of descriptors such as “mastery” and “full control” over one’s mental or brain states, of “instantiating” or “realising” them without interference, as well as the freedom to direct one’s consciousness. What are the assumptions and implications behind these descriptors? We will now consider some potential ways of thinking about them.

### Absolute Mental Control

There are numerous possible interpretations of the idea of being deprived of “full control” over one’s thoughts, or of controlling one’s own consciousness. One of these, which we take to be the least plausible, is that we usually have the ability, from the perspective of conscious experience, to organize and deliberately select our thoughts and the broader contents of our consciousness. Such an assumption, although not directly articulated in these terms, hints at the idea of complete mental control, as if one is at a control panel deciding which thoughts to engage with at any moment.

However, this seems an incorrect description of how thoughts, and mental content in the sense of ideas, emotions, and perceptions that form an individual's mental life, arise from one’s subjective standpoint. As a matter of conscious experience, we do not select thoughts as if from a menu of entertainment or deliberative options: thoughts *arise* for the most part unbidden in the mind [24]. Many can attest to the constant inane monologue playing out in the theatre of their mind, with regular unwanted, distracting, if not thoroughly unhelpful or distressing content occupying that seemingly automated process [25].

Even when an effort is made to intentionally deliberate about a specific topic, this seems to be at least in part much closer to a process of discovery (of discovering what thoughts will arise from entertaining certain other thoughts), rather than purely a process of *selecting* which specific thoughts to have [26, 27]. Indeed, any such wilful selection seems conceptually impossible: to *will* certain thoughts to occur to you at a given moment would require knowing the content of those thoughts *before* having those very thoughts – an impossible demand [28]. This is all the more obvious for mental states like beliefs or desires. While there is clearly some meaningful sense by which to describe our capacity to change our beliefs and attenuate our desires, we do not have the ability to select these à la carte: one cannot simply will the adoption of certain beliefs or desires in a given moment, in the sense of deciding to now truly believe the world is flat or that one feels genuinely satiated despite a pang of hunger the moment before.^2^

This is all to say we shall assume that any reference to depriving individuals of “full control” or “mastery” over their thoughts, mental states or consciousness (let alone the brain’s chemistry) cannot be employing this absolute sense of mental control, because it is a kind of control that we lack anyway. Of the ways of interpreting what is of concern to this literature, this cannot be it.

### Freedom From Mental Interference

More plausibly, the references to control among these commentators on mental integrity relate to a more modest interpretation: that of being free from others interfering with one’s thoughts or mental states. Hence, one could say that while we clearly cannot pick and choose which thoughts to have in a given moment, we can still meaningfully have control over them if they are determined by, or stem from, our own minds rather than from outside of them.

In other words, there may be an important sense of control that relies on assuming that the thoughts that occur to you, or the mental or brain states you have, appropriately stem from *you*, rather than someone or something else.^3^ One way to think about this is to consider two factories you might own. One produces specific items over which you lack immediate control, but where the raw materials on which they are based are determined by you. The other factory produces items over which you also lack immediate control, but where the raw materials are interfered with and chosen by others, and/or more directly where the items produced are interfered with by others (regardless of the raw materials). The latter scenario suggests an important loss of control over one’s factory, even if one never had “full” control over each item the factory produces anyway.

Is this what full control over one’s thoughts or mental states amounts to? While this notion of control does not assume the possibility of absolute subjective command over mind, it seems to change the central question from one of what counts as control in this context to what counts as “interference”. On the face of it, if interference just means influence of any kind, that seems too broad. This is because others have an influence on – that is, the power to affect – our mental states all the time. This can happen directly in a given moment, but also formatively. Directly, our mental states are influenced each time we speak to others, or each time we read some text. Thoughts, feelings, desires arise via others affecting our current mental states merely by interacting with us.

In some cases, those influences go on to be formative in that they change us more fundamentally in terms of our traits and dispositions. Reading certain “life changing” books and having certain intense conversations can be formative in that sense. More broadly the very process of socialisation – of learning to behave in ways deemed acceptable by one’s society – renders the idea of having thoughts or states of consciousness completely free from the interference of others impossible, and indeed undesirable. Which is to say, any broad notion of interference as mere influence seems an unpromising interpretation of what the nature of concerns about mental integrity amount to.

### Freedom From Harmful Mental Interference

Perhaps the worry is about a specific class of mental interference. There are several possible candidates, but a promising one might be limited to specific influences that are harmful. Which is to say, while interferences with one’s thoughts and consciousness by others and by one’s environment are unavoidable, the relevant sense of control here is being protected from interferences by others that leave us worse off in some sense.

However, what counts as being harmed or made worse off is difficult to settle. For instance, it cannot be that the concern over mental integrity comes down to being protected from *any* harmful mental interference. People interfere with our mental states all the time in ways that harm us on any plausible sense of that term, and in ways no one thinks we have a moral or legal right to be protected from. Imagine being sincerely told by a loved one that you are a disappointment. It is likely these words would interfere with your mental states in devastating ways. While it may be that it is rarely justified to tell that to someone, it does not follow that we have a right to be protected from others potentially telling us that. The same goes for other, every day instances that can harmfully interfere with our mental states: the news, advertisements, films, books, everyday interactions with friends or strangers – all these can give rise to thoughts, feelings, desires, beliefs that leave us worse off. Which is to say, there can be no plausible claim that we have a right to control our mental states or our consciousness in such a way as to be free from *any* harmful interference unless we were to conduct our lives in complete isolation. Such a right would be too demanding.

What about specific types of harm? Consider neurotechnologies that enable telepathy-like communication. Existing proof of concept examples involve relaying information from a sender to a receiver by magnetically stimulating the latter’s occipital lobe to trigger the perception of small bursts of light to communicate simply coded words like “hola” and “ciao” [19]. This kind of technology could potentially be used to relay harmful messages between individuals, but the fact that it is direct in the sense that it by-passes our sensory apparatus seems irrelevant on its own.

However, contrast this with a (more hypothetical) neurotechnology that enables ‘senders’ to alter the beliefs and desires, and more fundamentally the traits of those on the other side of the interface. This appears a qualitatively different kind of (potential) harm, as the recipient’s mental states are modulated without any conveyance of symbolic meaning that could be evaluated. This may be closer to the relevant way of interpreting concerns about mental integrity (we discuss such examples in the section ‘Bypassing rational control’). It is true that receiving harmful messages through such an interface could potentially alter someone's beliefs, desires or traits. However, this would represent an indirect influence on mental states, mediated through the interpretation of symbolic information. The recipient still has an opportunity to rationally evaluate the message and its implications. We explore this possibility in more detail below. For now, we only hope to have shown that if what is considered a violation of mental integrity is so broad that it includes any mental interference in the sense of influence, and *any* kind of harmful influence, this would provide a poor framework from which to evaluate the ethics of specific neurotechnologies that may threaten it. Not only would it render most examples of neurotechnologies as potential violations of mental integrity, but it would include much of human interaction as well.

### Control as Privacy

An alternative interpretation of what is meant by control in this context is a more mundane variety associated with the privacy and confidentiality issues raised by monitoring physiological data. For instance, in line with favouring a right to mental integrity, Ienca and Andorno [2] argue for “a right to brain privacy” that protects against illegitimate access to individuals’ neural data. Similarly, Lavazza [7] defines the mastery associated with having mental integrity to include our mental states and neural data not being non-consensually read, spread or altered in order to condition or influence the individual any way. A more recent paper by Lavazza and Giorgi [8] makes a stronger case, arguing for the uniqueness of mental privacy and its centrality to mental integrity.

The concern here is presumably this: if others have access to our neural data, they may have the power to use that information without our consent, potentially to our disadvantage. For instance, they might use it to infer facts about our preferences and dispositions, and potentially to manipulate us more effectively than standard ways of trying to alter people’s behaviour (e.g. education, advertisements, economic incentives, and so on). Or they might use it to limit certain opportunities that might otherwise be open to us (as in the context of paid insurance or employment), or to lower our social status. Lavazza and Giorgi [8] give an alternative example of an extremist religious group that uses technology to see if their captive really has converted to the group’s religion, or if the captive is just pretending to save themselves.

However, if that is all that the concern over mental integrity is, this would be much more straightforwardly an issue of informed consent when it comes to handling individuals’ neural data. It may be that there is an argument to be made for a specific legal protection, but morally, there is nothing unique here. In relation to brain-computer interfaces for instance, so long as users understand how their neural data will be used, and what the risks are, then their right to mental integrity in this sense is protected.

Is this what the moral significance of mental integrity amounts to when referring to control over mental states or brain data? It may be that some degree of mental privacy is necessary for mental integrity, but on its own this would be another implausible formulation of what mental integrity amounts to, at least if the concept is to remain etymologically tethered to the notion of having a coherent or whole mental life. Indeed, even in Lavazza and Giorgi’s [8] extremist example, there is no reason to believe that violating the mental privacy of the captive – wrong as it is – would in itself damage the coherence or wholeness of their mental life.

We mentioned earlier the bundling of concerns regarding mental states and brain or neural states in this literature. But concerns about control over one’s thoughts and consciousness are clearly distinct from these comparatively standard privacy issues about one’s data. It is one thing to lose control over one’s recorded neural data such that others can use it, it is another thing to have one’s mental states interfered with so that there is a loss or diminishment of control over its contents. Our goal remains to figure out what "control" means in that latter case.

### Direct Interference

Might the crux then be the *manner* by which some neurotechnologies interact with the brain? The directness of neurotechnologies certainly seems paramount to the debate. Consider this framework by Ienca and Andorno [2] for thinking about what qualifies as a threat to mental integrity: “For an action X, to qualify as a threat to mental integrity, it has to: (i) involve the *direct* access to and manipulation of neural signalling (ii) be unauthorized—i.e., must occur in absence of the informed consent of the signal generator, (iii) result in physical and/or psychological harm” (emphasis added).

Note how much work the term “direct” does here for thinking about threats to mental integrity. Without it, and as we have discussed in previous sections, mental integrity would be under constant threat since we clearly have (some forms of indirect) access to individuals’ neural signalling that can allow us to interfere with them without their consent, and in ways that harm them. Merely speaking to someone impacts their neural signalling if their brain is to process speech at all, and since we can infer various facts about others’ mental states and/or traits by observing their behaviours and expressions, we can threaten their mental integrity by speaking to them in ways we know will harm them (see Thornton and colleagues [29] for a distinction between mental states and traits).

Perhaps merely observing the behaviour of others can only tell us so much about their mental states (i.e. we might only be able to glean whether they are angry, sad or intoxicated), and perhaps our ability to interfere with aspects of their mental states will be limited via speech or any sensory stimuli. In that regard, neurotechnology does *not* pose a qualitatively different or unique threat to mental integrity, but a quantitative one: the directness might mean *more* power to interfere. And to that extent that this is true, there is a debate to be had about when that power is sufficiently strong to be morally distinct from other non-neurotechnological ways of interfering with them (we come back to this in the next section).

But note that this framework by Ienca and Andorno [2] includes not just actions with significant or overpowering threats to mental integrity but *any* action. There is no qualitative distinction. And without the caveat of *direct* access to, and manipulation of, neural signalling, such an account would be overly demanding and incompatible with the reality of everyday human interactions.

Moreover, it is not obvious what the moral significance of the directness of neurotechnologies amounts to in itself. There are two issues here. The first is the significance, if any, of intervening directly in the brain as opposed to intervening in the brain indirectly *via* the ears or any other sense organ. The second is identifying the relevant threshold for a brain intervention to be considered “direct”. Let us start with the latter.

Consider the various ways “direct” brain stimulation techniques work. Must the relevant neurons be in direct contact with the source of the stimulating electrode – i.e. must they be touching? If so, this would exclude techniques like transcranial magnetic and electric stimulation, which influence neurons indirectly through the skull. That would in turn exclude the bulk of neurotechnologies, including non-invasive brain-computer interfaces.

Perhaps ‘direct’ is intended to include anything that is part of the nervous system at all, or anything that influences the brain by means other than engaging perceptual processing [e.g. 26]. This would presumably include direct peripheral nerve stimulation techniques, which have been used for, among other things, reducing pain via electrodes placed near the vagus nerve to “trick” the brain into reducing painful signals [30]. Accounts that emphasize the “directness” of certain neurotechnologies – which, so far as we can tell, is all of them – owe us an account of what the relevant threshold is for something to be considered ‘direct’, otherwise it is unclear exactly which class of brain interventions is motivating their concerns.

Ultimately, even if there is a non-arbitrary threshold for directness to be identified, it remains ambiguous why it would be morally significant in a way that indirect interventions are not or need not be. For instance, there does not seem to be any morally relevant difference between stimulating the eyes to see something and directly stimulating the visual cortex to see the same thing, all else being equal.^4^ This brings us back to the first question posed in this section: what is the significance of directness, assuming we can identify it?

### Bypassing Rational Control

One answer to the question of the significance of directness is to claim that we have a greater capacity to assess information that comes to us via our senses, as opposed to stimuli that do not engage them. This is indeed what Bublitz and Merkel [1] argue for when discussing the relevance of the directness of neurotechnologies: “Persons have most control over interventions whose sensual substrates they perceive, particularly those rising to the level of conscious awareness. We can think about what we see and hear, we can modify and process it.”

This brings us back to trying to interpret the notion of control used in this debate, and gives a more obvious role for the idea of directness to play. It is this: the moral significance of neurotechnology’s directness is not so much that the electrode or whatever source of brain stimulation or recording is physically touching the nervous system, it is that it can influence certain mental states or traits without conscious, rational processing. In that sense, information that is relayed through our senses, as opposed to stimuli that bypass them, is subject to an important kind of control mechanism.. Information that is perceived through our senses provides us with an opportunity to recognize and reflect upon it. This process opens the door to influencing how this information integrates into our psychological framework. This is because there is an opportunity to deliberate, question, and challenge the information before it indirectly shapes our beliefs, desires, or traits.

Note however that, just because an intervention bypasses our senses does not mean it also averts our ability to perceive the information *as if* it is sensory information. In fact, many neurotechnologies involve transferring information via perception, even if not through the sense organs. This is especially the case with a brain-to-brain interface, which involves recording information from one brain and sending it to a computer, and then delivering that information to a receiving brain via some stimulation technique.

These often involve stimulating the motor cortex or occipital lobe of the receiver. For instance, Jiang and colleagues [20] set up a multi-person brain-to-brain interface, that involves three individuals collaborating to play a Tetris-like game. The one “receiver” is told by the two “senders” whether to rotate an incoming Tetris-like block by having their occipital lobe stimulated in such a way so that they perceive a small burst of light in their visual field. It is their perception of that spark that informs them of what to do – in fact, in this experimental set up, sometimes the senders intentionally relayed false signals, so not only could the receiver recognize and interpret the signal as if it were a sign in the outside world, they had to also consider its veracity.

On such a setup, it is not clear why this would be any different from playing that game vocally or using sign language. There is no reason to believe this bypasses the receiver’s rational capacities any more than traditional ways of hearing or seeing a signal (i.e. via the senses).

Which is to say, just because a neurotechnology transfers information in a way that bypasses our senses does not mean it also bypasses our capacity to rationally assess that information. The two can come apart, as described in Jiang and colleagues’ experiment [20]. This is an important specification, since a great deal of funding and hype when it comes to neurotechnologies is precisely for this kind of technologically-enabled telepathy – and so long as what is being telepathized is recognized by the recipient as incoming information or stimuli that is open to evaluation, it is unclear why it would undermine this kind of control associated with rationally processing input associated with the neuro-intervention, and therefore why it would be a threat to mental integrity if that is what it all boils down to.

Nevertheless, there are other ways of using neurotechnologies, including brain-to-brain interfaces, that could be direct in their reasoning-bypassing influence on mental states and traits, and which may involve a diminishment of the kind of control afforded by rational processing. An example of this may be the effects of DBS on mental states and personality traits, with reports of patients feeling alienated from their mental content (thoughts, ideas, emotions) while using it to treat Parkinson’s Disease [3, 23, 31, 32]. By alienated we simply mean an experience of separation from one’s mental content that may be distressing. To what degree DBS can cause this is controversial, and it may be that if patients understand that these impacts on their mental states are due to DBS, they may be able to evaluate and potentially moderate them over the long-term. However, to the extent that immediate feelings of alienation stem from direct changes that bypass certain levels of rational processing, then DBS presents an archetypal example of a neurotechnology that could threaten mental integrity.

On this picture, some neurotechnologies are “direct” in that they interfere with mental states and traits in a way that bypass the cognitive resources that might otherwise subject their input to some evaluative process associated with rationality. Indeed, it is precisely this bypassing potential of neurotechnologies that concerns other commentators writing about mental integrity [4, 33].

To make this bypassing claim clearer, consider the difference between these three scenarios: a) someone trying to persuade you the Earth is flat where you have the chance to hear them speak, interpret their words and assess the source/person they are coming from and the implications of that proposition (even if fleetingly); b) as above, but the words you hear do not come via soundwaves stimulating your ear drums but through stimulation of your auditory cortex, and c) having whatever relevant parts of your brain stimulated so that you come to think the Earth is flat without assessing the idea at all.

Only “c” depicts a scenario that captures this idea of direct mental interference where there is a clear loss of control in the sense of having at least the opportunity to check and monitor input via some rational process. This notion of control is by no means the kind of absolute mental mastery that involves picking and choosing what to think or believe at will – it is closer to a “quality control” system that processes information to check it broadly coheres with other beliefs, desires, values, and so on.^5^

To be clear, we do not invoke scenario “c” because we assume current neurotechnologies can crudely insert individual beliefs as if adding a new line of code into a program; our goal is only to depict a clear-cut example of a bypassing neurotechnology, and to distinguish it from other neurotechnologies that are “direct” in their impact on the nervous system but which do not bypass our rational capacities (i.e. scenario “b”).

Another way to think about this is to consider the difference between going on an arduous journey where some of your core beliefs and values are challenged so that by the end of it your worldview and personality are markedly changed, versus using brain stimulation techniques that instantiate similar changes. In the former, the end result is (at last in part) a product of processing and minimally evaluating one’s experiences while on that journey, while in the latter that evaluative process is circumnavigated.

To come back to the point of this section, which is to identify the significance of directness in this debate, we now have a clear answer. The threat to mental integrity posed by certain neurotechnologies is not that they stimulate the nervous system by going around the senses. It is that they stimulate the nervous system in a way that side-steps the opportunity to rationally evaluate their potential influence on mental states or traits, and thereby to control the degree to which they do in fact influence (or interfere with) them.

For instance, if while on that journey we observe a budding disposition to, say, talk to strangers despite historically being fairly introverted, we might have an opportunity to reflect on that and how this new tendency aligns with our values and other dispositions. The end result may therefore be one of endorsing our new disposition. Or not – we might find the new disposition comes with undesired trade-offs that mean we revert to our introversion fairly quickly. In contrast, directly modulating the brain so as to instil greater extroversion^6^ would not afford this reflective opportunity, and so risks that we might end up feeling alienated from this new, unendorsed disposition that now possibly sits inharmoniously with our values or other traits.

No doubt this bypassing capacity is not unique to just certain classes of neurotechnologies like DBS. Presumably we can describe (at least some of) the concerns associated with brain-washing, psychotropic drugs, and perhaps certain spiritual or near-death experiences, or simply from brain trauma due to an accident, along similar lines. These too can lead to sudden, radical changes in the mental states and traits of individuals, and at least part of the reason we find those changes potentially problematic is because there is a sense in which the person has lost an important kind of control that could have modulated the final outcome.

However, the fact that certain neurotechnologies are not unique in their potential to undermine this kind of control does not detract from having now homed in on what looks like the most plausible way to interpret the concern motivating some of this literature on neurotechnological threats to mental integrity. In the final section, we explore in more detail how to frame this concern in a way that is conducive to evaluating the ethics of specific neurotechnologies.

## Implications

What does the preceding discussion mean for mental integrity and neurotechnological threats to it? In trying to find the most plausible interpretation of some of the literature commenting on this issue, we have identified the idea that at least some neurotechnologies can be direct in their effect on our mental states or traits in the specific sense that they can bypass a form of control associated with the ability to rationally process stimuli.

If this is the most appropriate interpretation to help us evaluate neurotechnological threats to mental integrity, it may be tempting to dismiss the otherwise hazy concept of mental integrity and argue that the nature of this concern is best captured by reference to personal autonomy. If personal autonomy relates, at least in part, to the capacity of rationally modulating one’s beliefs and values, and mental integrity violations are instances of losing that kind of control, then there is a sense in which we do not even need this concept of mental integrity at all: we can simply refer to autonomy – for which there is a much more developed literature (see of relevance [34]) – in our attempts to capture what it is we are concerned about when we discuss specific neurotechnologies.

While this may be the parsimonious conclusion, jettisoning mental integrity and focusing explicitly on autonomy might nonetheless impoverish our ability to identify a particular subset of autonomy diminishment. As noted in the introduction, at least etymologically mental integrity relates to having one’s mental life be in some sense sound, whole and coherent – put another way, *not* disintegrated. In that light, reducing it to questions of personal autonomy misses out on something. This is because one may continue to consent to the use of a neurotechnology even if it has the effect of leaving one with the sense that some of their mental states now appear alien or distressingly separate from them. This might be because the neurotechnology has benefits that outweigh these costs, (e.g. by treating the symptoms of Parkinson’s [35]). In such a case, it may be that the neurotechnology in fact enhances overall autonomy even as it undermines mental integrity. It might enhance autonomy by enabling a person to pursue things they value more deeply despite coming at the cost of new, alien dispositions or mental content.

Moreover, many events or interventions that might undermine autonomy do not lead to any sense of alienation from our mental lives. For instance, our autonomy is undermined if we are not adequately informed about what we are consenting to – by not being informed, we are robbed of the opportunity to reflect on the implications of what we are signing up for, and so cannot evaluate how it aligns with our values. But nothing about that on its own entails a loss of mental integrity – our thoughts of indignation about being misinformed are compatible with having a whole, coherent mental life.

This suggests that if we want the concept of mental integrity to signal at something more precise than autonomy, we ought to home in not on all interventions that bypass rational control, but on specific ones: those that reliably lead to feeling alienated from one’s mental states. It is this particular variety of autonomy infringement that a focus on mental integrity can help us pick out.

It is important at this stage to recall our motivating goal: without a clear theory of mental integrity that explicates its constituent parts we are poorly positioned to weigh up what is morally at stake when evaluating specific neurotechnologies. If we understand threats to mental integrity very broadly as mental interference, we end up seeing those threats in neurotechnologies despite existing (for the most part unproblematically) in everyday interactions with others. On the other hand, if we understand these threats only as instances of bypassing rational control without necessarily including alienating effects on our mental lives, we end up equating it with any class of threats to autonomy, making the concept redundant.

To see this more clearly, it is worth revisiting Ienca and Andorno’s framework in light of our considerations so far. Recall that on their account, “for an action X, to qualify as a threat to mental integrity, it has to: (i) involve the *direct* access to and manipulation of neural signalling (ii) be unauthorized—i.e., must occur in absence of the informed consent of the signal generator, (iii) result in physical and/or psychological harm” [2].

Point (i) appears to give much significance to directness understood as the manner of stimulating the nervous system (i.e. not via the senses), which we have argued seems morally irrelevant on its own. (ii) is a straightforward autonomy concern and suggests it is impossible for there to be a loss of mental integrity in a context of informed consent. Yet, as with the example of DBS, we have good reason to disentangle consent from the effect of an intervention on our mental states or traits. And (iii) seems clearly too broad given the lack of specifics on the nature of the harm – what is the relevance of invoking mental integrity specifically if someone is using a brain-to-brain interface and receives a harmful message? This, on its own, seems a separate matter.

A much more useful role for the concept to play in ethical evaluation is to pick up a species of harm that is associated with the experience of alienation from our mental states or traits.^7^ Whether that harm results in a context of consent is, on its own, irrelevant: we want to be able to describe cases of consensual mental integrity diminishment as morally relevant despite the presence of consent. Similarly, whether it is direct in the sense of impacting neural signalling via the senses or not is irrelevant – as we have argued, bypassing the sense carries no special moral weight on its own.

## Conclusion

We have considered a number of ways of thinking about the relevance of mental integrity and how it might relate to ideas of control. We have found certain interpretations implausible, the foremost being that, but for the threat of certain neurotechnologies, we each possess absolute command over our mental lives that affords us “full control” or mastery over our thoughts, consciousness, mental or brain states. But similarly implausible, we have argued, is that the concern is about mental interference broadly construed as influence, including harmful influence of any kind. We also argued that while there may be relevant privacy concerns raised by neurotechnologies, these appear distinct issues: there is a meaningful role for the idea of control to play associated with privacy, but it is difficult to see how it relates to the mental integrity debate. Finally, we argued that while directness in terms of the immediate access a neurotechnology has to the nervous system seems in itself irrelevant, directness in the sense of interfering with mental states and/or traits in a way that bypasses our rational capacities does seem an important way of understanding threats to the wholeness or coherence of our mental lives. This brings concerns over mental integrity much closer to ones over personal autonomy, with threats to it being more precisely about interventions that bypass rational capacities in ways that reliably lead to alienation from one’s mental states or traits.

This discussion serves as a stepping stone for further explorations and is not intended to be a conclusive viewpoint on this complex topic. Future work should elaborate on the nature of the experience of being in some sense distressingly separate from some of one’s mental content due to a neurotechnological intervention, as well as qualify what amounts to strong and weak instances of it. This would include investigating which neurotechnologies are most likely to lead to such effects – all crucial endeavours but not ones we have aimed at here.

## Footnotes

1 See Kringelback and Deco on a framework for defining a ‘brain state’ and the associated challenges [36]. We thank a reviewer for bringing it to our attention.

2 The kind of control we have over our beliefs is a fraught philosophical debate, though even supporters of what is called doxastic volunteerism (the view that people have direct voluntary control over their beliefs) would not claim we can simply adopt a new belief *in a given moment* no matter how irrational we may perceive it to be [37].

3 This way of thinking about control is similar to the one deployed in debates on free will where an agent acts freely so long as they are the appropriate source of their actions [38]

4 To be clear, if stimulating the visual cortex involves invasive techniques that come with their own safety risks, that may be a relevant distinction. However, the relationship between neurotechnology and mental integrity is clearly not about identifying the former’s safety. Safety may well be hugely morally significant when it comes to neurotechnologies, but it is not in itself what motivates concerns about mental integrity.

5 It is also this kind of “quality control” afforded to us when information is relayed via our perceptual apparatus (be it via the senses or the brain’s perceptual systems) – the seeing or hearing of that information allows for the possibility of recognizing it and deliberating about it and thereby potentially modulating how it impacts our beliefs, desires or traits.

6 Which may indeed be an effect of DBS in some instances [32].

7 We consider such harms to be “pro tanto” harms, meaning that potential benefits that accompany that harm may create situations where violations of mental integrity are overall beneficial – as may be the case with modulating traumatic memories such that one becomes alienated from them (we thank a reviewer for raising this example). In other words, any harm in becoming alienated from one’s traumatic memories could be considered minor when compared to the harm caused by continuing to be traumatised by a memory (in itself a threat to mental integrity). Conversely, the benefit of becoming alienated from a traumatic memory—namely, the potential for reduced distress and improved mental health—could significantly outweigh the harm of the estrangement itself.
